# Adverse childhood experiences and behavioral problems in early adolescence: An empirical study of chinese children

**DOI:** 10.3389/fpsyt.2022.896379

**Published:** 2022-08-09

**Authors:** Yunfan Chen

**Affiliations:** College of Marxism, Hunan Normal University, Changsha, China

**Keywords:** adverse childhood experiences, children, behavioral problems, experience study, Chinese children

## Abstract

The aim of the present study was to examine the relationships between ACEs and behavioral problems of children in their early adolescence in Chinese society. Results from bivariate analyses of 2,910 Chinese children in early adolescence indicated that children begin to exhibit behavioral problems being related to the exposure of adverse childhood experiences (ACEs). Compared to those with 0 ACEs, children with 4 or more ACEs were 4.45 times (*p* < 0.001), 4.44 times (*p* < 0.001), 7.80 times (*p* < 0.001), 4.49 times (*p* < 0.001), and 6.63 times (*p* < 0.001) more likely to demonstrate hyperactivity, peer communication problems, pro-social problems, emotional problems and conduct problems, respectively. Rural children, children of mothers with low education, and boys were particularly likely to have been exposed to multiple categories of ACE. This study evidenced that there was a strong association between exposure to ACEs and behavioral problems in early adolescence in China.

## Introduction

Adverse Childhood Experiences (ACEs) are potentially traumatic events that occur in childhood, such as experiencing violence, abuse, or neglect, witnessing violence at home and having a family member attempt to commit suicide (1). They also include aspects of a child's environment that can undermine their sense of safety, stability, and bonding, such as growing up in a household with substance abuse, mental health problems, or instability owing to parental separation, or the incarceration of a parent, sibling, or other member of a household (1). The current large-scale study on the relationship between ACEs and adult health was initiated based on the collaboration between the US Centers for Disease Control and Prevention and the Kaiser Permanente Health Assessment Clinic in San Diego. According to the Center's original ACE questionnaire, 7 types of ACEs: 3 types of child abuse (psychological, physical, and sexual) and 4 types of family dysfunction (violent treatment of mother, living without family members, drug abuse, mental health patients or suicide, or imprisonment) were assessed. Subsequent ACE studies incorporated neglect and parental divorce or separation into the ACE index (2). A large number of studies have examined the relationship between ACEs and the risks of adult health issues. ACEs (such as child abuse and family dysfunction) that affect adult health problems (such as liver disease and depression) have been tested widely (3, 4). Numerous investigators have reported the link between ACE exposure and social and health problems, including cigarette smoking (5), autoimmune disease (6), depressive disorders (7), and use of psychotropic medications (8). Despite this growing body of literature, the proximal effects of ACEs on behavioral outcomes of early adolescence have been underestimated. The current study prospectively examine the relationships between ACEs and behavioral problems of nearly 3,000 children in their early adolescence in Chinese society.

### ACEs and child behavior research

There is a significant positive correlation between childhood psychological abuse and adolescent aggressive behavior (9). The experience of child abuse is directly related to the aggression behavior in a university (10). Child emotional abuse, poor parent-child relationship patterns, and a father's strict parenting behaviors, all act as risk factors that provoke the augment against adolescent borderline personality disorder. ACEs can also cause Internet addiction and suicide among adolescents (11). The number of risk factors is closely related to the development outcome of children. The mental health disorder risks of individuals with two or four risk factors are 4–10 times that of individuals with no risk factors (12). The analysis of existing studies shows that ACEs has a certain correlation with adolescents' emotion, hyperactivity and aggressive behavior. Children with behavioral problems (including emotional symptoms, conduct problems, hyperactivity, peer communication, and pro-social factors) are more likely to encounter risks during adolescence, including smoking (13), substance abuse (14), and obesity (15), etc.

Differences exist in the risk of adversity in families across levels of socioeconomic advantage. Children are more likely to be victims of child maltreatment if they come from low-income or single-parent households (16). Children in socioeconomically disadvantaged families will have greater exposure to ACE categories compared to those of higher socioeconomic advantage (17).

### Current research

Early adolescence can be described as a period of life, typically occurring between the ages of 10 and 15 years, in which the youth undergo rapid physical, cognitive, and social transformation (18). In recent years, children aged 12–14 are more likely to have behavioral problems in China. Chinese experts in psychology, nursing, and other disciplines have drawn inspiration from the CDC-Kaiser ACE's link between ACE exposure and a wide range of physical and mental health outcomes to a certain extent. However, one weakness in these studies is the reliance on data in a certain school or a given area. This limited reliance is prone to a recall bias and a measurement error. Subgroup difference analyses are rare, so less is known about whether ACEs can have similar effects on more socially and economically diverse subgroups. These literatures that have linked childhood adversity to a wide range of health problems in adulthood overlooks the proximal effects of ACEs by primarily focusing on outcomes in adulthood. The research on the proximal relation can provide support for the precise intervention of youth social work in advance, so as to avoid further deterioration of adverse effects.

## Methods

### Data source and quality control

The data used in this study are from the survey data of the “high risk family child protection system research” in 2017. Entrusted by the National Social Science Fund, the project is jointly carried out by Policy Research Center of the Ministry of Civil Affairs of the People's Republic of China and Hunan Normal University. The total survey sample is 2,862 district and county junior high school students in the whole nation. The survey adopts multi-stage cluster random sampling method, based on the number of students in the district and county. First, 50 districts and counties were selected from all districts and counties in China by PPS method. Then a junior middle school was selected from each county according to the systematic random method. Furthermore, a class was randomly selected from each junior high school in Grade One, Grade Two and Grade Three of junior high school. Finally, we selected three rows according to the position of students' seats in the class (usually there are 5–6 rows in each class). The students with informed consent in this class were investigated. The final sample of the survey includes 57 classes of Grade one, 52 classes of Grade Two and 41 classes of Grade Three in 20 provinces and cities. A total of 3,000 questionnaires were distributed, and 2,950 were returned. After strict screening and elimination of invalid questionnaires (e.g., child abuse measurement scales with over three missing values in each indicator measurement), a total of 2,910 valid questionnaires were collected. The proportion of cases with missing data is 19%; therefore, multiple imputation is used to deal with the missing values in the analysis samples. In this study, all variables used in the statistical model were included in the multiple imputation calculation model, and 24 independent data sets were obtained after imputing missing values, than this data set was statistical analysis, and finally the analysis results were summarized. All the descriptive statistics and model outputs in this paper are the results of missing value processing. The results of multiple interpolations are similar to those of direct deletion method.

To ensure the privacy of the respondents and to enhance the quality of investigation, the investigation team signed a confidentiality agreement with the schools and the respondents, and obtained the consent of the parents and children before collecting data. To ensure quality, the only those who were qualified to provide psychological consultation services were selected as investigators; the investigators underwent training on providing psychological and support services for children; the questionnaire was filled in the enclosed environment provided by the school in spare time and all completed questionnaires were collected by the investigators from the seat of the respondents with no interventions; and rigorous screenings were employed to remove questionnaires with missing values. The respondents are required to maintain a distance of 1 m between their positions when filling out the questionnaire. On the one hand, it is to protect the privacy of participants and prevent participants from being laughed at or labeled; On the other hand, it is also to obtain real data, so that the respondents can express their true situation and ideas in the context of respecting their privacy.

### Measurement

After the CDC-Kaiser and domestic research in China, ACEs including 5 types of child abuse and 11 types of family dysfunction faced by children in early adolescence (12–14 years), were investigated in this study. Child abuse comprises emotional, physical, and sexual abuse, and emotional and physical neglect. Family dysfunction covers divorce, witnessing domestic violence, parental disability, parental alcohol abuse, parental drug abuse, parental suicidal intention, parents leaving home, poor living environment, scolding, parental gambling, and parental criminal records.

### Child abuse

In this study, childhood trauma questionnaire (CTQ) was used to examine maltreatment (19). The Chinese version of the questionnaire has good reliability and validity, and is widely used to measure child abuse (20, 21). The CTQ was split into five factors (emotional, physical, and sexual abuse, and emotional and physical neglect). Each factor table covered five items. Each item adopted a 5-point Likert scale type score score (0 = “never,” 1 = “occasionally,” 2 = “sometimes,” 3 = “often,” and 4 = “always”) (19). The five factor scores for each item were summed and then converted into dichotomous variables (if the total score is 0, variable value will be defined as 0; if the total score is 1 or more, variable value will be defined as 1). The statistical results showed that the reliability and validity of the scale were less affected by the re evaluation. Emotional abuse was measured using five items, such as “someone in the family calls me ‘stupid guy' and ‘I think my parents hate me' (α = 0.775)”. Physical abuse was measured using five items, such as “someone hits me at home heavily,” and “I have to go to the hospital,” etc. (α = 0.793). Sexual abuse was measured using five items, such as “someone intimidates or tempts me to do sexual things with him/her” (α = 0.776). Emotional neglect was measured using five items, such as “someone in the family makes me feel unimportant” (α = 0.802). Physical neglect was measured using five items, such as “I am not eating enough,” and “I often wear dirty clothes” (α = 0.708). The scale can be used to measure the abuse that happened to the respondents before age 10.

### Family dysfunction

Family dysfunction refers to the functional failure of family in the process of care and education of children, which mainly includes: absence of family role (such as divorce of parents), incompetence of role (such as drug abuse of parents), rejection of role (such as guardians who cannot fulfill their parenting obligations in reorganizing families) and role conflict (such as families in the conflict of employment and work) (22). According to the situation in China, there are 11 types of family dysfunction. A total of 11 problems in children's growth (0 = “yes” and 1 = “no”) were measured. The questions focused on divorce, quarrels and fights (witnessing domestic violence), parental disability, parental alcohol abuse, parental drug abuse (narcotic drugs), parental suicidal intentions, parents leaving home with no news of their whereabouts, messy living environment, scolding, parental gambling, and parental criminal records. To ensure the validity and reliability of the measurement, On the one hand, the expert evaluation method is used to test the validity. Five experts were invited to judge the consistency between the questionnaire title and the original content scope. The five experts are Ling Hui (engaged in personality disorder research), Xiao Han Shi (engaged in child psychology research), Chen Dan (engaged in child abuse research), Ou Yang Yan Wen (engaged in domestic violence research) and Zhao Lan (engaged in drug addiction and abuse research). The results showed that the questionnaire had good content validity. On the other hand, using Alpha Reliability coefficient method to test reliability. Alpha Reliability coefficient is tested to be 0.682.

### Behavioral problems

Behavioral problems in children were measured using the student version of the Strengths and Difficulties Questionnaire (SDQ) (23). The questionnaire has been used widely in China and has good reliability and validity. Chinese scholars have tested the reliability and validity of the questionnaire and found that it satisfied the requirements of psychological measurement (24). The student version comprised five factors (including emotional symptoms, conduct problems, hyperactivity, peer communication, and pro-social factors). Each factor included 5 items, each of which adopted a level 3-level score (0 = “no compliance,” 1 = “part compliance,” and 2 = “full compliance”). There were 25 entries in all. Items 7, 11, 14, 21, and 25 had to be scored in reverse. The total score was converted into dichotomous variables. Whereas, 0 marked the normal level, 1 marked the abnormal level. The five factor calculation criteria included:

(1) 5 measurement items for emotional symptom factors, such as “I am often worried, unhappy, and heavy-laden” (α = 0.703), where 0–6 points represent the normal level (defined as 0) and 7–10 points indicate the abnormal level (defined as 1);
(2) 5 measurement items for conduct problem factors (e.g., “I am often angry, lose my temper, often argue with others, and lie”) (α = 0.683), where 0–4 points represent the normal level (defined as 0) and 5–10 points indicate the abnormal level (defined as 1);
(3) 5 measurement items for hyperactivity factors, such as “I cannot settle down,” “I am often restless and uneasy” (α = 0.736), where 0–6 points represent the normal level and 7–10 points indicate the abnormal level.
(4) 5 measurement items for peer communication factors (e.g., “I often stay alone, usually play by myself”) (α = 0.769), where 0–5 points represent the normal level (defined as 0) and 6–10 points indicate the abnormal level (defined as 1).
(5) 5 measurement items for pro-social factors (e.g., “I try to be friendly to others”) (α = 0.701), where 5–10 points represent the normal level (defined as 0), and 0–4 points indicate the abnormal level (defined as 1).

Behavioral problems measure the performance of respondents in the last week.

### Analytical strategy

This study used SPSS 22.0 for statistical analysis. It first conducted a descriptive analysis which presented the results between behavioral problems in early adolescence and the ACE factors in the overall samples. We estimated the ordinary least squares, and linear and logistic regressions to investigate various components of the associations between ACEs and behavioral problems in early adolescence. In the first set of regression models, we examined whether there was a positive association between the quantity of adverse exposures experienced and behavioral problems. Next, we examined whether there were differential associations between ACE exposure and child behavioral outcomes across registered residence, gender, and maternal education subgroups. In each regression, we tested the statistical equivalence of the coefficients for each variable across equations. Finally, we investigated the relationship between ACE categories and behavioral problems.

## Results

There are 1,083 students in the sample, accounting for 37.2%; 982 students in grade two, accounting for 33.7%; 845 students in grade three, accounting for 29.0%. There are 1,488 boys in the sample, accounting for 51.8%; 1,385 girls, accounting for 48.2%.Emotional neglect (39.76%) and abuse (35.36%), and physical abuse (26.38%) and neglect (25.37%) were the top four factors that influenced ACE incidence rates. Rural children had a higher ACE incidence than urban children. The incidence among children whose mothers had lower levels of education was higher than among children whose mothers had a higher level of education. Boys are more vulnerable to physical abuse, emotional and physical neglect. Girls are more vulnerable to emotional abuse, as seen in Table 1.

**Table 1:** Prevalence of adverse childhood experiences.

	**Full sample (%)**	**Household registration**		**Gender**		**Maternal education**		
		**Urban (%)**	**Rural (%)**	**Boys (%)**	**Girls (%)**	** < HS (%)**	**HS(%)**	**>HS(%)**
**Child abuse**								
Emotional abuse	35.36 (0.009)	33.09 (0.013)	37.44 (0.013)	32.21 (0.012)	38.44 (0.013)	38.62 (0.013)	34.97 (0.020)	28.24 (0.017)
Physical abuse	26.38 (0.008)	23.27 (0.011)	28.89 (0.012)	29.69 (0.012)	22.62 (0.011)	28.8 (0.012)	24.96 (0.018)	21.3 (0.015)
Sexual abuse	14.06 (0.007)	11.87 (0.009)	16.13 (0.010)	16.3 (0.010)	11.45 (0.009)	15.44 (0.009)	12.26 (0.014)	11.34 (0.012)
Emotional neglect	39.76 (0.009)	35.51 (0.013)	43.79 (0.014)	40.17 (0.013)	38.83 (0.013)	44.14 (0.013)	36.88 (0.020)	32.23 (0.017)
Physical neglect	25.37 (0.008)	21.04 (0.011)	28.8 (0.012)	28.02 (0.012)	22.37 (0.011)	28.84 (0.012)	21.16 (0.017)	20.79 (0.015)
**Family dysfunction**								
Divorce	13.65 (0.006)	13.49 (0.009)	14.46 (0.010)	14.96 (0.009)	12.26 (0.009)	14.18 (0.009)	11.09 (0.013)	13.29 (0.013)
Witnessing of domestic violence	6.00 (0.005)	4.91 (0.006)	7.77 (0.007)	6.28 (0.006)	6.44 (0.007)	7.84 (0.007)	4.66 (0.009)	4.86 (0.008)
Parental disability	5.00 (0.004)	3.18 (0.005)	6.42 (0.007)	4.32 (0.005)	5.07 (0.006)	6.3 (0.006)	2.94 (0.007)	2.3 (0.006)
Parental alcohol abuse	7.00 (0.005)	4.19 (0.005)	9.25 (0.008)	7.83 (0.007)	5.35 (0.006)	8.96 (0.007)	4.33 (0.008)	3.92 (0.007)
Parental drug abuse	1.00 (0.002)	0.94 (0.003)	0.96 (0.003)	1.42 (0.003)	0.58 (0.002)	1.13 (0.003)	0.69 (0.003)	0.95 (0.004)
Parental suicidal intention	2.00 (0.003)	2.02 (0.004)	1.7 (0.004)	2.16 (0.004)	1.66 (0.003)	1.93 (0.004)	1.04 (0.004)	2.16 (0.005)
Leaving home for parents	1.00 (0.002)	1.23 (0.003)	1.41 (0.003)	1.28 (0.003)	1.45 (0.003)	1.46 (0.003)	1.21 (0.005)	1.08 (0.004)
Poor living environment	2.00 (0.002)	1.52 (0.003)	2.14 (0.004)	1.96 (0.004)	1.59 (0.003)	1.79 (0.003)	1.38 (0.005)	1.49 (0.004)
Scolding	11.00 (0.006)	7.51 (0.007)	13.28 (0.009)	12.54 (0.009)	8.24 (0.007)	13.92 (0.009)	8.12 (0.011)	5.41 (0.008)
Parental gambling	7.00 (0.005)	3.61 (0.005)	9.32 (0.008)	7.22 (0.007)	5.79 (0.006)	8.96 (0.007)	5.87 (0.010)	2.03 (0.005)
Parental criminal records	1.00 (0.002)	0.65 (0.002)	1.03 (0.003)	0.81 (0.002)	0.87 (0.002)	0.93 (0.002)	1.04 (0.004)	0.54 (0.003)
**Behavior issues**								
Emotional problems	4.71 (0.004)	4.78 (0.006)	4.59 (0.006)	3.55 (0.005)	5.84 (0.006)	4.98 (0.006)	3.83 (0.008)	4.63 (0.008)
Hyperactivity	5.05 (0.004)	4.71 (0.006)	5.25 (0.006)	6.1 (0.006)	3.84 (0.005)	5.65 (0.006)	5.66 (0.010)	3.57 (0.007)
Conduct problems	7.99 (0.005)	7.61 (0.007)	8.44 (0.008)	10.44 (0.008)	5.29 (0.006)	8.39 (0.007)	6.82 (0.011)	7.54 (0.010)
Peer communication problems	7.23 (0.005)	6.65 (0.007)	7.7 (0.007)	8.61 (0.007)	5.63 (0.006)	8.02 (0.007)	4.51 (0.009)	7.13 (0.010)
Pro-social problems	9.54 (0.005)	6.31 (0.007)	12.51 (0.009)	13.05 (0.009)	5.82 (0.006)	11.85 (0.008)	7.64 (0.011)	6.11 (0.009)

*N = 2,910*.

*HS, High School Percentages and means (standard errors in parentheses) are presented*.

The results showed that 28.2% of children had not suffered any adverse experiences (including 5 types of child abuse and 11 types of family dysfunction); 71.8% had suffered adverse experiences, of which 23.4% faced 1 ACE, 18.4% faced 2 ACEs, 11.9% faced 3 ACEs, and 18.1% faced 4 to 16 ACEs. Except for physical and emotional abuse (*r* = 0.573 *p* < 0.01), emotional and physical neglect (*r* = 0.618, *p* < 0.01), the correlation coefficients between each adversity factor were below 0.5, and there was only a weak correlation between the factors. The results of the total sample analysis revealed that ACEs were significantly associated with adolescent conduct, peer communication, and pro-social problems (Table 2). Compared to those with 0 ACEs (the reference group), children with 1 ACE had roughly 1.77 times the odds of demonstrating the level of child conduct problems (*p* < 0.05), children with 2 ACEs had 3.55 times the odds (*p* < 0.001), children with 3 ACEs had 6.04 times the odds (*p* < 0.001), and children with 4 or more ACEs had 6.63 times the odds (*p* < 0.001). Similarly, Children with 1 ACE had roughly 1.69 times the odds of demonstrating the level of peer communication problems (*p* < 0.05), children with 2 ACEs had 2.46 times the odds (*p* < 0.01), children with 3 ACEs had 1.80 times the odds (*p* < 0.05), and children with 4 or more ACEs had 4.44 times the odds (*p* < 0.001) when compared to children who had never faced any ACEs. Children with 1 ACE had roughly 2.64 times the odds of demonstrating the level of pro-social conduct problems (*p* < 0.01), children with 2 ACEs had 4.83 times the odds (*p* < 0.001), children with 3 ACEs had 6.33 times the odds (*p* < 0.001), and children with 4 or more ACEs had 7.80 times the odds (*p* < 0.001) when compared to children who had never faced any ACEs. The emotional problems and hyperactivity exhibit a similar positive and increasing association, although the influence of ACE exposure is not as strong. Compared to children with 0 ACEs, children with 3, and 4 or more ACEs had 2.75 times (*p* < 0.01) and 4.49 times (*p* < 0.001) the odds, respectively, of having emotional problems. Similarly, among children who faced 3, and 4 or more ACEs, the incidence probability of hyperactivity was 3.95 times (*p* < 0.001) and 4.45 times (*p* < 0.001), respectively, when compared to children who had never faced any ACEs.

**Table 2:** Logistic regression models estimating the association of ACEs on behavioral problems from 12 to 14 years old.

	**Emotional problems**	**Hyperactivity**	**Conduct problems**	**Peer communication problems**	**Pro-social problems**
	**Odds ratio (95%CI)**	**Odds ratio (95%CI)**	**Odds ratio (95%CI)**	**Odds ratio (95%CI)**	**Odds ratio (95%CI)**
**Panel 1: full sample**					
(0)					
1	0.76 (0.00, 1.57)	1.51 (0.79, 2.88)	1.77 (0.96, 3.26)^*^	1.69 (1.00, 2.87)^*^	2.64 (1.51, 4.61)^**^
2	1.22 (0.61, 2.43)	1.66 (0.85, 3.25)	3.55 (2.01, 6.29)^***^	2.46 (1.46, 4.13)^**^	4.83 (2.83, 8.26)^***^
3	2.75 (1.46, 5.19)^**^	3.95 (2.11, 7.38)^***^	6.04 (3.40, 10.72)^***^	2.80 (0.97,3.35)^*^	6.33 (3.63, 11.03)^***^
4+	4.49 (2.61, 7.71)^***^	4.45 (2.51, 7.89)^***^	6.63 (3.89, 11.32)^***^	4.44 (2.75,7.18)^***^	7.80 (4.66, 13.05)^***^
Constant	0.03^***^	0.02^***^	0.03^***^	0.03^***^	0.03^***^
**Panel 2: household registration**					
**Urban registered residence**					
(0)					
1	0.80 (0.29, 2.23)	1.08 (0.42, 2.78)	1.97 (0.83, 4.67)^*^	2.82 (1.25, 6.35)^*^	3.23 (1.23, 8.49)^*^
2	1.82 (0.75, 4.44)	1.65 (0.66, 4.12)	5.26 (2.40, 11.51)^***^	4.27 (1.91, 9.54)^***^	6.76 (2.69, 16.99)^***^
3	3.68 (1.49, 9.05)^**^	4.16 (1.72, 10.05)^**^	6.51 (2.77, 15.28)^***^	1.19 (0.32, 4.46)	10.44 (3.99, 27.33)^***^
4+	6.38 (2.97, 13.72)^***^	5.55 (2.54, 12.12)^***^	9.35 (4.32, 20.23)^***^	9.03 (4.16, 19.59)^***^	9.22 (3.63, 23.38)^***^
Constant	0.02^***^	0.01^***^	0.02^***^	0.02^***^	0.01^***^
**Rural registered residence**					
(0)					
1	0.65 (0.21, 2.00)	1.64 (0.63, 4.30)	1.53 (0.64, 3.64)	1.13 (0.54, 2.39)	1.83 (0.90, 3.71)^*^
2	0.63 (0.19, 2.13)	1.28 (0.44, 3.71)	2.21 (0.95, 5.14)^*^	1.49 (0.71, 3.13)	2.93 (1.47, 5.83)^**^
3	1.84 (0.70, 4.87)	3.16 (1.23, 8.08)^**^	4.42 (1.99, 9.86)^***^	1.68 (0.78, 3.63)	3.45 (1.72, 6.95)^***^
4+	3.28 (1.45, 7.44)^**^	3.50 (1.47, 8.34)^**^	4.29 (2.02, 9.14)^***^	2.46 (1.28, 4.74)^**^	5.34 (2.84, 10.02)^***^
Constant	0.03^***^	0.03^***^	0.03^***^	0.05^***^	0.05^***^
**Panel 3: gender**					
**Boys**					
(0)					
1	1.06 (0.26, 4.27)	1.61 (0.71, 3.65)	1.67 (0.77, 3.63)	1.62 (0.82, 3.19)	2.60 (1.30, 5.20)^**^
2	2.35 (0.68, 8.12)	1.92 (0.84, 4.39)	3.82 (1.87, 7.80)^***^	2.24 (1.15, 4.37)^*^	4.22 (2.13, 8.33)^***^
3	1.92 (0.48, 7.79)	3.39 (1.50, 7.66)^**^	4.54 (2.16, 9.56)^***^	0.79 (0.30, 2.07)	6.60 (3.32, 13.14)^***^
4+	7.00 (2.36, 20.76)^***^	2.63 (1.20, 5.77)^**^	5.79 (2.92, 11.51)^***^	3.78 (2.03, 7.05)^***^	7.24 (3.78, 13.87)^***^
Constant	0.01^***^	0.03^***^	0.04^***^	0.05^***^	0.04^***^
**Girls**					
(0)					
1	0.77 (0.32, 1.87)	1.37 (0.44, 4.29)	1.82 (0.63, 5.31)	1.51 (0.63, 3.61)	2.80 (1.04, 7.55)^*^
2	1.02 (0.42, 2.46)	1.18 (0.33, 4.24)	3.07 (1.10, 8.56)^*^	2.58 (1.13, 5.92)^*^	6.38 (2.52, 16.13)^***^
3	3.82 (1.81, 8.06)^***^	5.37 (1.91, 15.06)^***^	9.31 (3.56, 24.34)^***^	3.56 (1.48, 8.59)^**^	5.45 (1.94, 15.29)^***^
4+	4.58 (2.37, 8.84)^***^	8.30 (3.32, 20.72)^***^	8.47 (3.40, 21.06)^***^	4.81 (2.24, 10.30)^***^	9.01 (3.63, 22.32)^***^
Constant	0.04^***^	0.02^***^	0.02^***^	0.03^***^	0.02^***^
**Panel 4: maternal education**					
**< HK**					
(0)					
1	0.92 (0.34, 2.50)	2.44 (0.86, 6.94)^*^	1.66 (0.69, 4.02)	1.10 (0.57, 2.15)	1.43 (0.71, 2.86)
2	0.97 (0.35, 2.71)	2.26 (0.76, 6.69)^*^	2.79 (1.20, 6.48)^*^	1.26 (0.64, 2.47)	3.22 (1.69, 6.11)^***^
3	1.75 (0.66, 4.61)	6.02 (2.20, 16.52)^***^	5.27 (2.32, 12.01)^***^	1.33 (0.64, 2.77)	3.51 (1.79, 6.86)^***^
4+	3.95 (1.78, 8.76)^***^	5.58 (2.11, 14.74)^***^	5.35 (2.45, 11.66)^***^	2.25 (1.23, 4.10)^**^	4.27 (2.31, 7.89)^***^
Constant	0.03^***^	0.02^***^	0.03^***^	0.06^***^	0.05^***^
**HK**					
(0)					
1	0.36 (0.07, 1.76)	1.55 (0.46, 5.18)	1.82 (0.56, 5.85)	1.72 (0.38, 7.81)	3.35 (1.03, 10.94)^*^
2	0.27 (0.03, 2.25)	2.01 (0.57, 7.15)	2.46 (0.73, 8.30)	3.41 (0.80, 14.63)^*^	3.09 (0.85, 11.26)^*^
3	1.62 (0.40, 6.51)	4.13 (1.14, 14.94)^*^	4.05 (1.12, 14.65)^*^	1.26 (0.13, 12.40)	10.28 (3.00, 35.17)^***^
4+	2.37 (0.83, 6.77)^*^	3.86 (1.25, 11.89)^**^	3.78 (1.23, 11.66)^*^	4.90 (1.23, 19.43)^*^	7.99 (2.55, 25.10)^***^
Constant	0.04^***^	0.03^***^	0.03^***^	0.02^***^	0.02^***^
**>HK**					
(0)					
1	0.90 (0.16, 4.96)	0.26 (0.03, 2.10)	1.44 (0.38, 5.45)	3.48 (1.14, 10.59)^*^	15.10 (1.87, 121.92)^**^
2	3.95 (1.13, 13.73)^**^	0.94 (0.24, 3.70)	6.16 (2.15, 17.69)^***^	6.21 (2.16, 17.83)^***^	28.21 (3.63, 219.52)^***^
3	8.72 (2.54, 29.90)^***^	1.71 (0.43, 6.78)	8.92 (2.94, 27.05)^***^	1.58 (0.30, 8.33)	25.13 (2.97, 212.38)^**^
4+	11.67 (3.53, 38.51)^***^	5.18 (1.81, 14.86)^**^	15.47 (5.38, 44.46)^***^	18.02 (6.36, 51.08)^***^	41.59 (5.17, 334.67)^***^
Constant	0.02^***^	0.03^***^	0.02^***^	0.02^***^	0.01^***^

*N = 2,910*.

* *p < 0.05*.

** *p < 0.01*.

*** *p < 0.001*.

These subsequent regression models investigate whether there were differences in the associations between ACE exposure and behavioral outcomes across children's area of residence gender, and level of maternal education. The results of the analysis suggested the following. First, the high exposure to ACE increases the probability of hyperactivity, and emotional, conduct, peer communication, and pro-social problems among children living in urban areas. Compared to those with 0 ACEs (the reference group), the odds of urban children with four or more ACEs demonstrating high levels of hyperactivity, and conduct, peer communication, and pro-social problems was 6.38 times (*p* < 0.001), 5.55 times (*p* < 0.001), 9.35 times (*p* < 0.001), and 9.22 times (*p* < 0.001), respectively. Compared with rural children who did not have ACEs, the probability of emotional problems among rural children with four or more ACEs was 3.28 times (*p* < 0.01), hyperactivity was 3.50 times (*p* < 0.001), conduct problems was 4.29 times (*p* < 0.001), peer communication problems was 2.46 times (*p* < 0.01), and pro-social problems was 5.34 times (*p* < 0.001). Second, the high exposure of ACE increases the probability of girls' hyperactivity and conduct, peer communication, and pro-social problems. Compared with girls without any adverse experiences, for girls with 4 or more ACE factors, the probability of hyperactivity was 8.30 times (*p* < 0.001), conduct problems was 8.47 times (*p* < 0.001), peer communication problems was 4.81 times (*p* < 0.001), and pro-social problems was 9.01 times (*p* < 0.001). Third, the high exposure to ACE increases the probability of emotional, conduct, peer communication, and pro-social problems among children whose mothers had a junior college or higher educational background. Compared with children without any adverse experiences whose mothers had a junior college or higher educational background, the probability of emotional problems among children with 4 or more ACE factors was 11.67 times (*p* < 0.001), conduct problems was 15.47 times (*p* < 0.001), peer communication problems was 18.01 times (*p* < 0.001), and pro-social problems was 41.59 times (*p* < 0.001).

Our final set of regressions examined the relative association of each specific type of adverse event with behavioral problems after controlling for covariates for the full sample (Table 3). All results were correlated with more than one ACE. Although particular ACEs were found to have larger associations with behavioral outcomes when compared to others, associations between each ACE and outcome were not nearly as large as the associations found between cumulative ACEs and behavioral problems.

**Table 3:** Logistic regression models estimating associations of specific types of ACEs experienced with behavioral problems from 12 to 14 years old.

	**Emotional problems**	**Hyperactivity**	**Conduct problems**	**Peer communication problems**	**Pro-social problems**
	**Odds ratio (95%CI)**	**Odds ratio (95%CI)**	**Odds ratio (95%CI)**	**Odds ratio (95%CI)**	**Odds ratio (95%CI)**
Emotional abuse	2.35 (1.50, 3.67)^***^	2.09 (1.38, 3.16)^***^	1.56 (1.09, 2.23)^**^	1.15 (0.79, 1.66)	1.02 (0.73, 1.41)
Physical abuse	0.94 (0.77, 1.16)	0.97 (0.80, 1.19)	1.14 (0.97, 1.34)^*^	1.08 (0.91, 1.28)	1.10 (0.95, 1.28)
Sexual abuse	1.32 (1.08, 1.61)^**^	0.93 (0.74, 1.18)	1.34 (1.14, 1.58)^***^	1.14 (0.95, 1.37)	0.98 (0.82, 1.17)
Emotional neglect	1.18 (0.77, 1.82)	1.41 (0.93, 2.12)^*^	1.20 (0.85, 1.70)	1.26 (0.89, 1.80)	2.41 (1.75, 3.31)^***^
Physical neglect	1.48 (0.95, 2.29)^*^	1.47 (0.97, 2.23)^*^	1.41 (0.99, 2.00)^*^	1.29 (0.89, 1.87)	1.90 (1.39, 2.60)^***^
Divorce	0.88 (0.49, 1.58)	0.93 (0.54, 1.61)	1.22 (0.80, 1.88)	1.13 (0.73, 1.76)	1.48 (1.02, 2.13)^*^
Witnessing of domestic violence	1.57 (0.84, 2.90)	0.99 (0.50, 1.96)	1.51 (0.90, 2.54)^*^	1.40 (0.80, 2.43)	1.35 (0.82, 2.23)
Parental disability	0.94 (0.42, 2.12)	1.03 (0.45, 2.33)	1.05 (0.54, 2.03)	1.35 (0.71, 2.56)	1.20 (0.66, 2.17)
Parental alcohol abuse	1.18 (0.61, 2.27)	1.46 (0.79, 2.67)	1.38 (0.82, 2.34)	1.06 (0.60, 1.88)	0.77 (0.44, 1.36)^*^
Parental drug abuse	0.32 (0.05, 2.10)	0.78 (0.13, 4.55)	0.52 (0.12, 2.14)	1.95 (0.61, 6.23)^*^	1.98 (0.65, 6.05)
Parental suicidal intention	2.09 (0.82, 5.34)^*^	1.45 (0.49, 4.35)	1.39 (0.59, 3.31)	1.25 (0.50, 3.13)	0.99 (0.41, 2.40)
Leaving home for parents	1.77 (0.57, 5.52)	0.52 (0.10, 2.80)	0.87 (0.29, 2.63)	1.19 (0.41, 3.43)	1.40 (0.54, 3.63)
Poor living environment	1.48 (0.53, 4.18)	0.49 (0.12, 1.98)	0.95 (0.37, 2.43)	1.37 (0.55, 3.40)	2.57 (1.17, 5.64)^*^
Scolding education	1.64 (0.97, 2.76)^*^	1.79 (1.08, 2.96)^*^	1.76 (1.15, 2.69)^**^	1.48 (0.94, 2.34)^*^	1.33 (0.88, 2.03)
Parental gambling	0.81 (0.40, 1.64)	1.18 (0.64, 2.19)	0.71 (0.40, 1.27)	1.14 (0.66, 1.98)	0.65 (0.37, 1.17)^*^
Parental criminal records	0.29 (0.03, 3.04)	3.00 (0.88, 10.22)^*^	1.30 (0.37, 4.60)	1.04 (0.28, 3.87)	2.22 (0.73, 6.73)
Constant	0.02^***^	0.03^***^	0.02^***^	0.04^***^	0.04^***^
Observations	2,910	2,910	2,910	2,910	2,910

* *p < 0.05*.

** *p < 0.01*.

*** *p < 0.001*.

## Discussion

Several studies have focused on the relationship between ACEs and adult health issues, whereas very few ones have focused on ACEs and behavioral problems in early adolescence. Based on existing research, this study took 16 factors to build a measure of ACEs. Statistics have suggested that the incidence of ACE was high, and 71.8% of children have faced at least one ACE. In other studies in China, the proportion of children who faced at least one ACE was 66.22% (25). The CDC-Kaiser ACE study found that 64% of American children faced at least one ACE, by studying the retrospective data from adults (6). Some studies have also concluded that 75% of American children have faced at least one ACE based on children's 5-year-old sequence data (2). The top four common adverse experiences in this study are emotional neglect (39.76%), emotional abuse (35.36%), physical abuse (26.38%), and physical neglect (25.37%), which are consistent with the top three types of emotional neglect (26.65%), emotional abuse (24.25%), and physical neglect (21.52%) identified by Nie Junyan in China (25). The ACE factors and proportion of children in the current study are uniformed with those in the existing research. The main conclusions of this study are as follows.

We examined whether children exposed to ACEs in early adolescence were associated with behavioral problems. We found that there was a strong association between exposure to ACEs and behavioral problems in early adolescence. The accumulation of ACE factors has a “threshold effect” on early childhood problems (26). Studies have shown that after the accumulation of ACE factors, especially 3, 4, or more, the probability of early childhood behavioral problems will multiply. Compared with children who faced no ACEs, the probability of conduct problems in children with 4 or more factors was 6.04 times, of peer communication problems was 4.44 times, of pro-social problems was 6.33 times, of hyperactivity was 4.45 times, and of children's emotional problem was 4.49 times. The possibility of behavioral problems among children aged between 12 and 14 years with 4 or more ACE factors is 4–6 times that of children without any adverse experiences. To the best of our knowledge, our study is the first to investigate the relationship between ACE factors and behavioral problems among children aged between 12 and 14 years.

Investigation of subgroup differences indicated that children living in urban areas are less likely to suffer from adversity than those living in rural areas. Children whose mothers have higher levels of education are less likely to suffer from adversity than those whose mothers have lower levels of education. Girls are less likely to suffer from adversity than boys. However, children living in urban areas, those whose mothers have higher education levels and girls with high exposure to ACEs have a higher probability of facing problems in their early youth, which keeps constant with the conclusion drawn by **(author?)** (2) that American children whose mothers have high school or higher levels of education are more likely to show behavioral problems after exposure to ACEs. According to the differential susceptibility hypothesis, Children have differential susceptibility to rearing environments' influence. Some children are not only more vulnerable than others to the negative effects of adversity, but also to the beneficial effects of a rich upbringing environment (27). The interaction between children and their surroundings is shaping individual susceptibility. Inferior environment may reduce individual susceptibility, while superior environment may also improve individual susceptibility. Children living in rural areas with less educated mothers may be at a greater social and economic disadvantage, but some of them can develop their resilience in adversity, making them less vulnerable to behavioral problems. Some people are is not only more vulnerable to the positive impact of the positive environment, but also more vulnerable to the negative impact of the adverse environment. Children who live in urban areas and whose mothers have a higher level of education have less social and economic disadvantages, but are more sensitive to problems and more vulnerable to impact. Once they are highly exposed, children living in urban areas and whose mothers have high levels of education will have a higher probability of behavioral deviation. Gender differences are associated with the learning environments of boys and girls. Boys' academic performance in school is usually worse than that of girls, creating what is known as the “boy crisis”. Boys are growing up with less-involved fathers and are more likely to drop out of school, drink, be addicted to drugs, become delinquent, and end up in prison (28). Boys are more influenced by peer culture. When their peers do not agree with the academic performance, boys are more likely to get anti-school attitudes and behaviors (29). Girls generally have good academic performance, but they also face greater peer competition pressure. These growing environments make it easier for boys to become a low susceptibility group and girls to become a high susceptibility group. Girls are more sensitive than boys, and are more inclined to have problems when exposed. To the best of our knowledge, this is the first study to investigate differences in the relation of ACEs across household registration subgroups.

Though an exploratory study was conducted on the proximal effects of ACEs in early adolescence, the following shortcomings remain. First, the retrospective research design was limited. Compared with the longitudinal study design, data reliability and validity obtained through retrospective research in this study were relatively insufficient. Although there was no significant difference in grade and age, the difference between parents (and their children) who chose to participate and not participate was not considered. Second, the independent variables were hardly exhausted. According to the existing research and domestic conditions, ACE factors are expanded to 16, and there may be unmeasured variables related to ACE behavior problems. Other adversities in the CDC-Kaiser study include personal victimization, financial hardship, and discrimination. Future research should examine a wider range of ACEs that are probably associated with different populations, leading to poor health or behaviors (30). Third, the existing studies provide limited explanations for the formation of stress resistance. This study focused on the interpretation of ACEs with respect to early childhood behavioral problems. The positive explanatory power for ACEs was limited. However, according to relevant research, in the tracking study of 698 cases of victims born in poverty, stress, abuse, and neglect conditions, 2/3 were found to be well-functioning adults (31).

Despite these shortcomings, this study has made a vital contribution to the existing literature on adverse situations faced by children. By analyzing the proximal effects of ACE exposure, this study has extended the range of ACE effects. The results indicated that children in early adolescence begin to show behavioral problems after exposure to ACEs. Finally, to understand how these groups are affected by ACEs in China, the observations of previous ACE studies were extended to populations in urban and rural areas, of different genders, and with different maternal educational backgrounds.

## Data availability statement

The original contributions presented in the study are included in the article/supplementary material, further inquiries can be directed to the corresponding author.

## Ethics statement

The studies involving human participants were reviewed and approved by Biomedical Research Ethics Committee of Hunan Normal University. Written informed consent to participate in this study was provided by the participants' legal guardian/next of kin. Written informed consent was obtained from the individual(s), and minor(s)' legal guardian/next of kin, for the publication of any potentially identifiable images or data included in this article.

## Author contributions

The author confirms being the sole contributor of this work and has approved it for publication.

## Funding

Research on Child Abuse Prevention System in High Risk Families was funded by the Youth Project of National Philosophy and Social Science Fund in China (Project No. 12CSH095).

## Conflict of interest

The author declares that the research was conducted in the absence of any commercial or financial relationships that could be construed as a potential conflict of interest.

## Publisher's note

All claims expressed in this article are solely those of the authors and do not necessarily represent those of their affiliated organizations, or those of the publisher, the editors and the reviewers. Any product that may be evaluated in this article, or claim that may be made by its manufacturer, is not guaranteed or endorsed by the publisher.
